# Correlation between Soluble *α*-Klotho and Renal Function in Patients with Chronic Kidney Disease: A Review and Meta-Analysis

**DOI:** 10.1155/2018/9481475

**Published:** 2018-08-12

**Authors:** Qinglian Wang, Wenyan Su, Zhenwei Shen, Rong Wang

**Affiliations:** ^1^Department of Nephrology, Shandong Provincial Hospital Affiliated to Shandong University, Jinan, China; ^2^Qilu Pharmaceutical Co., Ltd., Jinan, China

## Abstract

**Objective:**

Over decades, numerous inconsistent studies are reported on the relationship between soluble *α*-Klotho and renal function in patients with chronic kidney disease (CKD). This study aims to perform a meta-analysis to figure out the correlations between soluble *α*-Klotho and renal function in patients with CKD.

**Materials and Methods:**

We searched medical and scientific literature databases, PubMed and EMBASE (from the inception to October 2017), for publications that reported studies on associations between soluble *α*-Klotho and renal function in patients with CKD. Only publications in English were extracted. Summary correlation coefficient (r) values were extracted from each study, and 95% confidence intervals (CIs) were calculated. Publication bias was tested, and sensitivity and subgroup analyses were performed to investigate potential heterogeneity.

**Results:**

Of 611 studies, 9 publications with 1457 patients were included into the analysis. The following data were extracted from the literature: first author, year of publication, research region, research index, sample size, average age and Pearson or Spearman correlation coefficient, study design, the *α*Klotho/FGF23 assays utilized, full length, or the C-terminal fragment of FGF23. The pooled r between *α*-Klotho and estimated glomerular filtration rate (eGFR), FGF-23 were 0.35 (95%CI, 0.23~0.46, and P<0.05), -0.10 (95%CI, -0.19~-0.01, and P<0.05) with remarkable significance, indicating moderate heterogeneity. There was no significant heterogeneity between subgroups in analyses of *α*-Klotho and eGFR stratified by research region, mean age, and eGFR, but heterogeneity exists in analyses of *α*-Klotho and FGF-23 stratified by research region. There was no significant correlation between a-klotho and Ca and PTH and PHOS. There was no evidence of publication bias with Egger's test (p=0.360) or with Begg's test (p=0.902) and the distribution of funnel plots was symmetrical in all of our analysis.

**Conclusions:**

There exists a significant positive correlation between soluble *α*-Klotho and eGFR in patients with CKD. Also, a significant negative correlation between *α*-Klotho and FGF23 levels is proven. This raises hope to employ *α*Klotho and FGF23 as early biomarkers of CKD. However, further large prospective follow-up researches are needed to validate this hypothesis and to explore whether maintaining or elevating the Klotho level could improve renal function and complications in CKD patients.

## 1. Introduction

The *α*-Klotho was originally identified as an aging suppressor by Kuro-M et al. in 1977, the year when they found the Klotho-deficient mice displayed phenotypes resembling human premature-aging syndrome with shortened lifespan [1]. The gene encoding a single-pass transmembrane protein is mainly expressed in distal convoluted renal tubules, choroid plexus of the brain [1] and parathyroid [2] and also in pituitary gland [3], inner ear [4], breast epithelial cell [5], and other tissues [1]. As an obligatory coreceptor of fibroblast growth factor 23(FGF23), it forms a complex with fibroblast growth factor receptor (FGFR) [6, 7], which can maintain mineral homeostasis by regulating urinary phosphorus reabsorption and inhibiting production of 1,25-dihydroxy vitamin D in kidneys and suppressing synthesis and secretion of parathyroid hormone in parathyroid gland [8]. In addition to its membrane-bond form, there is also a soluble form generated by proteolytic release of the transmembrane form or alternative transcript splicing [9, 10]. As a humoral regulator, it can be detected in plasm, urine, and cerebrospinal fluid. And it has pleiotropic biologic effect including inhibiting the signal transduction pathway of insulin/insulin-like growth factor 1 and Wnt, reducing oxidative stress as well as regulating nitric oxide synthesis [11].

CKD is considered to be an increasing public health issue with low awareness rate and its prevalence rate is about 8-16% around the world [12]. The leading cause of morbidity and mortality in patients with CKD is cardiovascular diseases (CVD) which cause great financial burden [13]. In recent years, as kidney function declines, these risk factors such as abnormal calcium-phosphorus metabolism, oxidative stress, and anemia have become conspicuous and ulteriorly increase cardiovascular diseases risks [14]. As these abnormal indexes which could be detected at early stages of CKD predict CKD progression and adverse outcomes, they could be applied as early biomarkers. Hence, identification of CKD at early stages is critical to monitor disease progression and reduce cardiovascular collapses.

In recent years, emerging evidence suggests that the serum soluble *α*-Klotho could serve as an early biomarker for CKD. It has been reported that serum level of soluble *α*-Klotho decreased in the early stage of CKD and there was a significant inverse correlation between soluble *α*-Klotho level and kidney function decline [14–16]. However, some studies have yield contradictory results that soluble Klotho levels were not pertinent to renal function [17–19]. Thus, we conducted a meta-analysis by summarizing the available evidence in order to overall assess the diagnostic value of serum soluble Klotho level for CKD.

## 2. Materials and Methods

### 2.1. Literature Search

Two researchers independently searched the PubMed (MEDLINE included) and EMBASE library databases from inception to October 2017 for related published studies. Our research was limited to literatures written in English. Index terms we used to search the indicated databases were ((Chronic Kidney Diseases) OR (Chronic Renal Diseases) OR (Chronic Kidney Insufficiency) OR (Chronic Renal Insufficiency) OR (CKD ) OR (Chronic nephropathy) OR (Chronic kidney failure) OR (Chronic Renal failure)) AND ((Klotho protein) OR (KL protein) OR (alpha-Klotho protein) OR (*α*KL)). Secondary references included in these literatures were also recruited.

### 2.2. Study Identification and Selection

First papers without detailed data and duplicates in terms of CKD and Klotho protein were excluded. Thereafter 611 publications were involved into further analysis. Two independent reviewers evaluated the potentially relevant articles on the basis of the inclusion and exclusion criteria as shown in Table 1.

**Figure 1:**
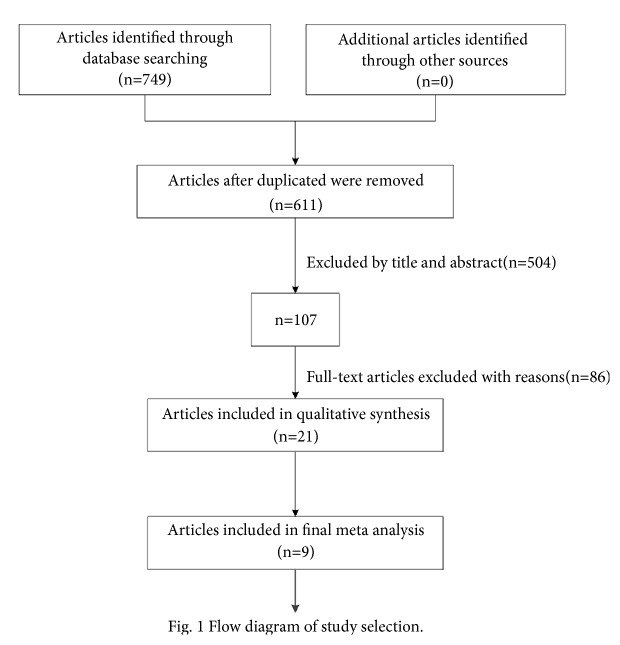
Flowchart of the study selection process.

If there was discordance among the 2 independent researchers for one study, its eligibility was decided by the 3rd investigator. Overall 504 publications were excluded by looking through title and abstract. Then 86 publications were excluded by reading the full articles; therefore 21 publications were reserved. Further by reading thoroughly and discussing together, 12 publications were excluded. Finally 9 publications with 1457 patients were comprised. 9 patients' samples were included. Detailed information about flowchart of the study selection process was shown in Figure 1.

### 2.3. Quality Assessment

For quality assessment of each included study, we considered items in the Strengthening the Reporting of Observational Studies in Epidemiology (STROBE) guidelines [20]. We focused particularly on the following main characteristics of the study, including the study design and participants' characteristics in the statistical analysis. As there is no consensus on the tools to evaluate the quality of observational cross-sectional studies, this article did not give a comprehensive score. Moreover, a simple application of such scores was discouraged in previous studies [21–23].

### 2.4. Data Extraction

The data were extracted from the included literatures by two investigators (Qinglian Wang and Wenyan Su) independently, and the extracted contents included the following: first author, year of publication, research region, research index, sample size, average age and Pearson or Spearman correlation coefficient, study design, assay utilization about FGF23 and *α*-Klotho, and intact/C-terminal fragment of FGF-23 measured. Spearman and Pearson correlation coefficients were all recorded in different articles. In order to utilize data better, we converted the published Pearson correlation coefficients into Spearman correlation coefficients as the latter was not affected by logarithmic transformation [24]. Furthermore, since the sampling of Spearman correlation coefficient is not normally distributed and the confidence interval (CI) was calculated depending on the value of correlation coefficient. We converted each correlation coefficient to an approximately normally distributed z value by Fisher's transformation and then calculated the standard error (SE) of z. The z value was then converted by inverse Fisher's transformation to obtain correlation coefficient and CI. A third reviewer joined to assess the terms and vote for a decision when disagreement occurred.

### 2.5. Meta-Analysis

After Fisher's z~r transformation, random effects meta-analysis was chosen to combine the data [25]. Heterogeneity was obtained by calculating the Q value and inconsistency index (I2) [26]. P<0.05 or I^2^>50% indicated the existence of heterogeneity. Sensitivity analysis was performed when notable heterogeneity existed to further explore the source of heterogeneity. Studies were stratified by following grouping methods: (1) patients age (<60y or ≥60y), (2) research region (Asia or Europe), and (3) eGFR (≥45ml/(min·1.73m^2^) or <45 ml/(min·1.73m^2^)).

Publication bias was assessed by Begg's and Egger's test and visually assessed by funnel plot.

All the statistical analyses were carried out using Stata Software (Version 13.0 StataCorp, College Station, TX, USA).

## 3. Results

### 3.1. Study Characteristics

We included 9 associated studies from 9 publications in our systematic review and meta-analysis (Table 2). Of them, 8 publications [14–18, 27–29] reported data on *α*-Klotho and eGFR, with 1136 participants. 7 publication [14–16, 18, 19, 27, 29] reported data on *α*-Klotho and FGF23, with 1239 participants. 4 publications [14, 15, 19, 27] reported data on *α*-Klotho and Ca, with 863 participants. 3 publications [14, 15, 19] reported data on *α*-Klotho and PTH, with 678 participants. 5 publications [14, 15, 19, 27, 29] reported data on *α*-Klotho and PHOS, with 887 participants (Table 2).

The 9 publications were published between 2012 and 2014. 5 publications were from the AISA and 4 from Europe. All of the 9 studies were conducted in both male and female (Table 2).

### 3.2. Results of Meta-Analysis

#### 3.2.1. *α*-Klotho and eGFR

**Figure 2:**
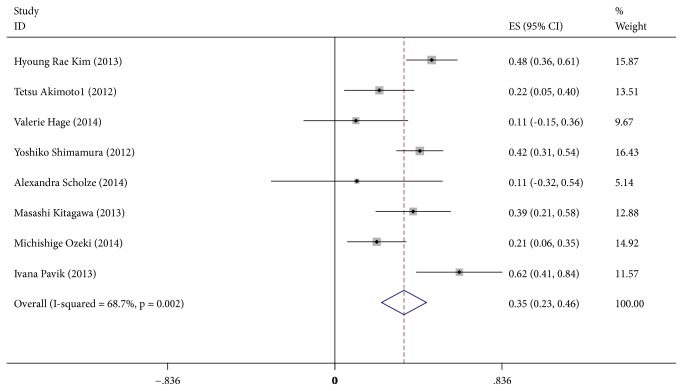
Forest plots of the summary correlation coefficient (r) with corresponding 95% CIs for the correlation between *α*-Klotho and eGFR in patients from all eligible studies.

Figure 2 shows the meta-analysis of the correlation between *α*-Klotho and eGFR. All of the included 8 studies showed a positive correlation between *α*-Klotho and eGFR. There was a significant heterogeneity among the 8 studies (I^2^=68.7%, P=0.002), so we used a random effects model. The pooled Spearman correlation coefficient was 0.35 (95%CI, 0.23~0.46, and P<0.05), suggesting that there was a significant positive correlation between *α*-Klotho and eGFR.

There was no evidence of publication bias with Egger's test (p=0.360) or with Begg's test (p=0.902). The distribution of funnel plots was symmetrical (Supplementary Materials Figure 1). The result is suggestive of an indistinctive small study bias. In the sensitivity analysis, the pooled RR ranged from 0.32 (95%CI, 0.19~0.44) to 0.37 (95%CI, 0.26~0.48) (Supplementary Materials Figure 2). No study had a significant impact on the total combined results.

To explore potential sources of moderate heterogeneity across studies and to examine the impact on final summary estimates, we conducted a series of subgroup analyses according to mean age (<60y or ≥60y), research region (Asia or Europe), eGFR (≥45ml/(min·1.73m^2^), or <45 ml/(min·1.73m^2^)). There was no significant heterogeneity between subgroups in analyses of *α*-Klotho and eGFR stratified by research region, mean age and eGFR (P for group heterogeneity>0.05). Positive correlations were apparent in all subgroups, and all of them were statistically significant (Supplementary Materials Table 1).

### 3.3. *α*-Klotho and FGF-23

**Figure 3:**
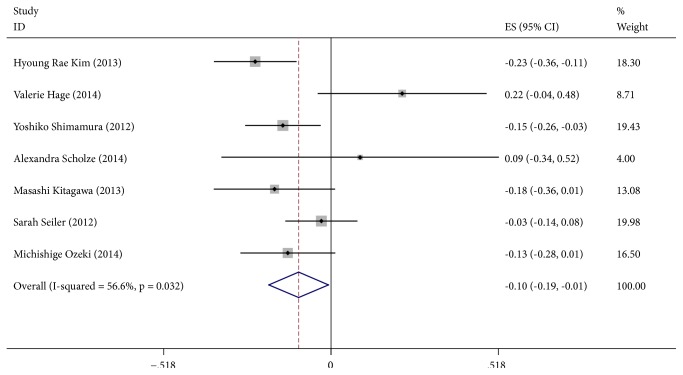
Forest plots of the summary correlation coefficient (r) with corresponding 95% CIs for the correlation between *α*-Klotho and FGF-23 in patients from all eligible studies.

Figure 3 (*α*-Klotho and FGF-23) shows the meta-analysis of the correlation between *α*-Klotho and FGF-23. Among the included 7 studies, 5 found a negative correlation between *α*-Klotho and FGF-23 and 2 found a positive correlation. With a random effects model (I^2^=56.6%, P=0.032), the pooled Spearman correlation coefficient was -0.10 (95%CI, -0.19~-0.01, and P<0.05), suggesting that there was a significant negative correlation between *α*-Klotho and FGF-23.

There was no evidence of publication bias with Egger's test (p=0.781) or with Begg's test (p=1.000). The distribution of funnel plots was symmetrical (Supplementary Materials Figure 3). The result is suggestive of an indistinctive small study bias. In the sensitivity analysis, the pooled RR ranged from -0.13 (95%CI, -0.20~-0.06) to -0.07(95%CI, -0.17~-0.02) (Supplementary Materials Figure 4). No study had a significant impact on the total combined results.

To explore potential sources of moderate heterogeneity over studies and to examine the impact on final summary estimates, we conducted subgroup analysis according to mean age (<60y or ≥60y), research region (Aisa or Europe), and eGFR (≥45 ml/min·1.73m^2^ or <45 ml/min·1.73m^2^). There was no significant heterogeneity between subgroups in analyses of *α*-Klotho and FGF-23 stratified by mean age and eGFR (P group heterogeneity>0.05). Research regions might be the sources of heterogeneity (P group heterogeneity<0.05) and participants in Asia subgroup showed a significant negative correlation between *α*-Klotho and FGF-23, while participants in Europe subgroup showed no correlation. Negative correlations between *α*-Klotho and FGF-23 were apparent in age and eGFR subgroups, and all of them were statistically significant (Supplementary Materials Table 1).

### 3.4. *α*-Klotho and Ca, PTH and PHOS

**Figure 4:**
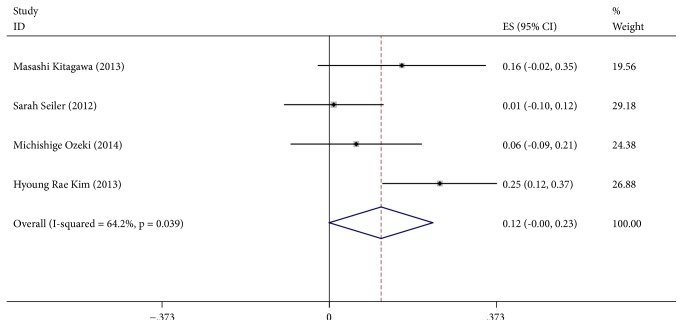
Forest plots of the summary correlation coefficient (r) with corresponding 95% CIs for the correlation between *α*-Klotho and Ca in patients from all eligible studies.

**Figure 5:**
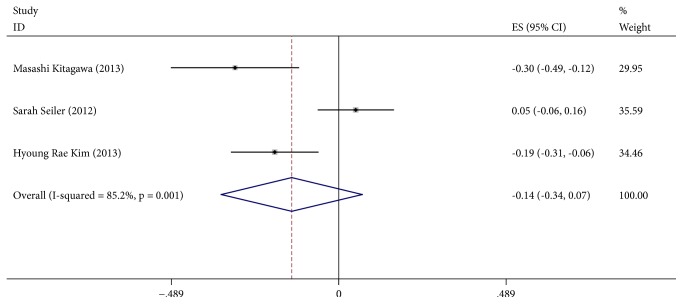
Forest plots of the summary correlation coefficient (r) with corresponding 95% CIs for the correlation between *α*-Klotho and PTH in patients from all eligible studies.

**Figure 6:**
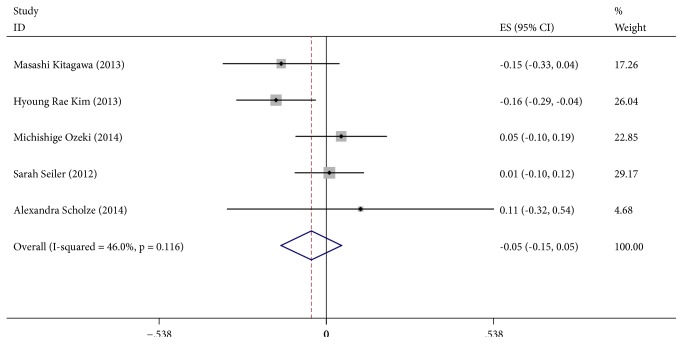
Forest plots of the summary correlation coefficient (r) with corresponding 95% CIs for the correlation between *α*-Klotho and PHOS in patients from all eligible studies.

Figure 4 (*α*-Klotho and Ca) shows the meta-analysis of the correlation between *α*-Klotho and Ca. All of the included 4 studies found a positive correlation between *α*-Klotho and Ca. With a random effects model (I^2^=64.2%, P=0.039), the pooled Spearman correlation coefficient was 0.12 (95%CI, 0.00~0.23, P>0.05), suggesting no significant positive correlation between *α*-Klotho and Ca.  Figure 5 (*α*-Klotho and PTH) shows the meta-analysis of the correlation between *α*-Klotho and PTH. We included 3 studies, 1 found a positive correlation between *α*-Klotho and PTH, and 2 reported a negative correlation. With a random effects model (I^2^=85.2%, P=0.001), the pooled Spearman correlation coefficient was -0.14 (95%CI, -0.34~0.07, P>0.05), suggesting no significant correlation between *α*-Klotho and PTH.  Figure 6 (*α*-Klotho and PHOS) shows the meta-analysis of the correlation between *α*-Klotho and PHOS. We included 5 studies, 3 of them found a positive correlation between *α*-Klotho and PHOS, and 2 reported a negative correlation. With a random effects model (I^2^=46.0%, P=0.116), the pooled Spearman correlation coefficient was -0.05 (95%CI, -0.15~0.05, P>0.05), suggesting no significant correlation between *α*-Klotho and PHOS. Egger's test or with Begg's test show no publication bias. The distribution of funnel plots was symmetrical (Supplementary Materials Figures 5, 7, and 9). The result is suggestive of an indistinctive small study bias. In the sensitivity analysis, the pooled RR ranged from 0.05 (95%CI, -0.03~0.13) to 0.16 (95%CI, 0.04~0.28) (Supplementary Materials Figure 6) of Ca, the pooled RR ranged from -0.22 (95%CI, -0.33~-0.12) to -0.07 (95%CI, -0.30~0.17) (Supplementary Materials Figure 8) of PTH, and the pooled RR ranged from -0.08 (95%CI, -0.19~0.08) to -0.00 (95%CI, -0.09~0.08) (Supplementary Materials Figure 10) of PHOS. No study had a significant impact on the total combined results.

## 4. Discussion

Kidney is a major organ to maintain soluble Klotho homeostasis by two ways. One is to cleavage membrane-bound *α*-Klotho in the renal tubular epithelial cells and release into circulation. And the second is to eliminate redundant and unnecessary soluble *α*-Klotho from circulations into the urinary lumen through renal proximal tubules by transcytosis to play its biological role [30]. In recent years, the correlation between soluble *α*-Klotho and renal function in patients with CKD has been intensively studied [14, 15, 17, 18, 31]. A group of studies showed that there existed a sustained *α*-Klotho deficiency status in patients with CKD and soluble *α*-Klotho levels gradually became lower as eGFR declined [32–34]. In elderly well functioning adults, there also exists a longitudinal association between low soluble Klotho and decline in kidney function by a large, diverse cohort [35]. These findings raise hope to employ *α*-Klotho as an early biomarker for renal function decline in patients with CKD. However, contrary to what was mentioned above, some studies found that soluble *α*-Klotho levels had no significant correlation with eGFR [17–19] or even two studies reported *α*-Klotho were higher in patients with CKD [31, 36], we performed this review and meta-analysis to assess the correlation between soluble *α*-Klotho levels and renal function in patients with CKD.

In this meta-analysis, with a random effects model, we found a significant positive correlation between soluble *α*-Klotho and eGFR in patients with CKD as the pooled Spearman correlation coefficient was 0.35 (95%CI, 0.23~0.46). And the subgroup analysis revealed that there was no significant heterogeneity between subgroups including age, study area, and eGFR, which suggests that soluble *α*-Klotho level declines as renal function decreases independent of age, study area, and eGFR. This conclusion was in accordance with previous studies [14–16]. As *α*-Klotho is expressed in multiple tissues, the strongest expression by far is in kidney and also experimental data has showed that kidney directly contributes to circulating *α*-Klotho [30]. It can be surmised that if kidney injuries, fibrosis occurs and renal function declines, its production would decrease (including membrane *α*-Klotho and soluble*α*-Klotho) [15], which has been proved by a prior study that the level of mRNA expression and protein production of *α*-Klotho both reduced significantly in the kidneys among patients with chronic renal failure [34]. And a recent study demonstrated the serum soluble *α*-Klotho levels significantly decreased after retroperitoneoscopic nephrectomy in 2 days and 5 days in living donors, respectively [37]. Furthermore, as for CKD patients, uremic toxins like indoxyl sulfate or p-cresyl sulfate induce hypermethylation of CpG dinucleotides in the *α*-Klotho gene and inhibit *α*-Klotho expression [38, 39]. These findings suggested that *α*-Klotho has obvious correlation with renal function in multiple studies.

As a humoral regulator, *α*-Klotho exerts many effects including antiaging, antioxidative stress, and antiapoptosis [40, 41]. In experimental models, there has been ample evidence proving that *α*-Klotho has a protective effect against kidney injury by gene transfer. Sugiura et al. found that Ad-kl (adenovirus harbouring the mouse Klotho gene) gene transfer ameliorated apoptosis attenuated tubular damage and reduced serum creatinine in ischaemic acute renal injury [42]. And Mitani et al. proved that Ad-kl gene transfer can ameliorate morphological kidney damage and improve creatinine clearance in angiotensin II-induced renal damage [43]. Similar findings were also seen in an immune-complex glomerulonephritis model [44]. Hence, it could be speculated that as kidney function declines, the production of *α*-Klotho decreases and, due to lack of the protective effects of *α*-Klotho, the kidney function deteriorates further, thus forming a vicious cycle. So it can be concluded that *α*-Klotho might act as a prospective factor for renal function decline and replacement therapy may act as a crucial method. But large prospective follow-up cohorts are expected to conduct to validate our conclusions.

Conceivably, restoring *α*-Klotho levels by reactivating endogenous expression of *α*-Klotho gene or providing exogenous *α*-Klotho might be a novel therapeutic target for patients with CKD [45]. And some rodent animal experiments have already proved that bolus supplementation of soluble Klotho protein is safe and effective for protecting kidney function and retarding CKD progression [46–48]. Experiments on primates or even clinical experiments are in urgent needs.

In our meta-analysis, we also found a significant negative correlation between *α*-Klotho and FGF23 levels in CKD patients. With a random effects model (I^2^=56.6%, P=0.032), the pooled Spearman correlation coefficient was -0.10 (95%CI, -0.19~-0.01, and P<0.05). The same conclusions were also made by other studies [16, 49–51]. On the one hand, as soluble *α*-Klotho is mainly generated by proteolytic release of the transmembrane form in kidney, soluble *α*-Klotho is a reflector of renal *α*-Klotho expression [8] and the two forms of *α*-Klotho would decrease together when the renal function declines. As a coreceptor for FGF23, a decreased expression of transmembrane *α*-Klotho would contribute to a compensatory increase in FGF23 to maintain mineral homeostasis [19]. So in early stage of CKD, serum phosphate is not elevated. With kidney function deteriorating, *α*-Klotho further decreases and exacerbates FGF23 resistance. And the elevated FGF23 in turn decreases *α*-Klotho expression [52], thus forming a vicious cycle. Finally the elevated FGF23 levels can no longer compensate for dietary phosphorus overload which contributes to overt hyperphosphatemia in advanced stages of CKD. On the other hand, soluble *α*-Klotho can inhibit sodium phosphate cotransporters in the proximal tubules, thus promoting urinary phosphate excretion [10]. A decrease in soluble *α*-Klotho may result an increase in serum phosphate levels due to increased phosphate absorption, which in turn can stimulate FGF-23 production. However, Isakova et al. [51] found there was a subtle decrease in serum phosphate in the early stages of CKD. Meanwhile, levels of FGF23 and fractional excretion of phosphate (FEPi) increased and 1,25D levels decreased. They inclined to that these syndromes were in accordance with primary FGF23 excess rather than primary *α*-Klotho deficiency. Because if Klotho deficiency is the initial factor that causes secondary increases in FGF23 secretion, the subtle signs of Klotho deficiency would be manifested in the early stage of CKD, such as increases in serum phosphate, 1,25D and calcium levels. However, the observed syndromes were contrary to what was mentioned above. And there is an alternative, unifying explanation that increases in FGF23 is the initial factor causing secondary downregulating of *α*-Klotho by hypophosphatemia or reduced 1,25D3 [53]. And Tamara Isakova et al. [51] supposed that maybe by increasing expression of a stimulus for FGF23 secretion or decreasing production of an inhibitor, the kidney injury itself is an initial stimulus for FGF23 increasing in early CKD. Its possibility is supported by the animal studies: when kidney was injured, FGF23 rose immediately,which precedes any increase in serum phosphate [54]. To date, it is still unclear which one of FGF-23 upregulation and *α*-Klotho deficiency occurs first.

By the subgroup analysis, we found that the correlation varied as study area differed; for example, participants in Asia subgroup demonstrated a significant negative correlation between *α*-Klotho and FGF23, while participants in Europe subgroup demonstrated no statistical correlations. The following ideas may, to some extent, explain the differences: firstly, the genetic differences exist among different races, which may influence the expression of soluble *α*-Klotho and FGF23; secondly, there is only 3 publications included in Europe subgroup, and maybe we can get significant result by increasing the sample size; thirdly, as mentioned above, the levels of serum phosphate are a stimulus for FGF23. And the serum phosphate levels among different countries may be variable due to dietary phosphorus intake, medication, socioeconomic status, and other factors. For example, it has been reported that there is an independent association between poverty and higher serum phosphate levels [55, 56]. We supposed that participants in Europe may have a better control of serum phosphorus and that the level of FGF23 is relatively lower, which needs to be validated by further clinical study.

In this analysis, we also figured out the correlations between soluble *α*-Klotho and serum phosphorus, PTH, and calcium respectively, but failed to confirm its correlations statistically. As an autocrine or paracrine enzyme, soluble *α*-Klotho reduces urine phosphorus reabsorption by inhibiting the expression of renal sodium phosphate cotransporter NaPi-2a [10] and increases urine calcium uptake by stabilizing TRPV5 calcium channel [57] independent of FGF23. It is reported that parathyroid resistance, an increase of FGF23 failing to decrease parathyroid hormone levels, is mediated, in part, by depressed expression of *α*-Klotho [58–60]. It can be speculated that soluble *α*-Klotho can decrease serum phosphate and PTH levels as well as elevate serum calcium levels. But it has been reported that there was a significant inverse correlation between serum Ca and soluble *α*-Klotho in Children with CKD [61]. And among most patients with mild-to-moderate CKD, serum phosphate levels are normal due to compensatory increases in FGF23 and PTH [49, 62]. And *α*-Klotho is only one of the factors regulating mineral metabolism. The levels of serum phosphorus, PTH, and calcium are influenced by so many other factors such as transmembrane *α*-Klotho, soluble *α*-Klotho, FGF23, 1,25D3, dietary, and medication and these factors are interacting with each other by complicated ways. We cannot completely eliminate the effects of these factors on our analysis.

**Figure 7:**
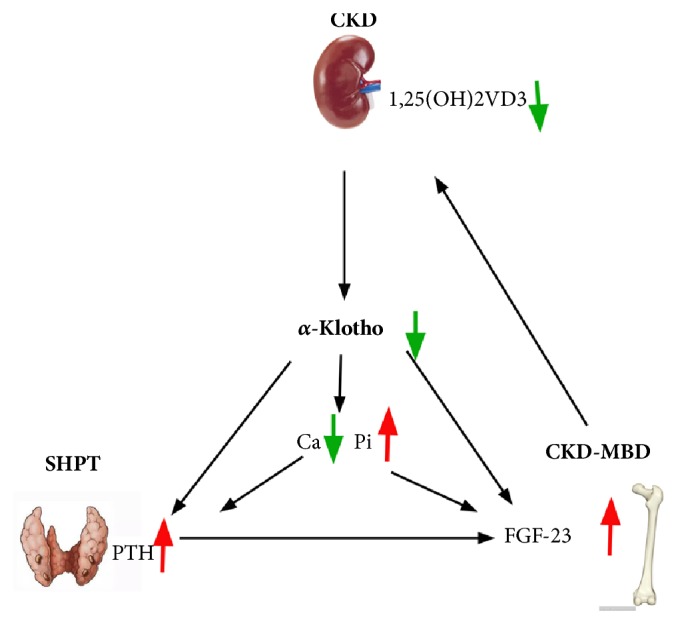
Cross connection diagram of hypothesis.

In summary, as kidney function declines, soluble *α*-Klotho decreases and FGF23 increases. But it is still unclear which one of them occurs first. At its early stages, these changes could help maintain mineral homeostasis. With kidney function deteriorating, the suppressive effect of increased FGF23 on serum phosphate and PTH production is defunctionalized due to furtherly decreased *α*-Klotho. As we could see, hyperphosphatemia and secondary hyperparathyroidism (SHPT) occur. And 1,25D3 decreases when kidney function declines, due to it is produced in kidney. Also, it can be suppressed by increased FGF23, which leads to decreased serum calcium. Low calcium and hyperphosphatemia further exacerbate SHPT and elevate PTH and FGF23 levels. Changes above form a vicious cycle and in concert contribute to the development of complications of CKD, such as metabolic bone disease, SHPT and vascular calcification (Figure 7). 

*Limitations*. Our study still has some inherent limitations. Firstly, although the total number of involved patients in our meta-analysis is relative large, the number of the participants in some of the included studies is quite small. In this case, the conclusions in them would be less convincing. Secondly, our analysis is based on studies that had been published, which tends to reveal positive and significant results. Some studies with negative and insignificant results might be rejected. Moreover, some results associated with our analysis are still on the way to be accepted. This would cause publications bias. Thirdly, in some clinical researches and basic researches, they revealed positive or negative correlation between these indexes, but no R values or raw data were supplemented to calculate correlation coefficient. Fourthly, our meta-analysis is restricted to articles published in English, which would lead to publications bias. However, the results of Begg's test showed no evidence of publication bias. We also used the random effects model to reduce heterogeneity. Therefore, the results of the present study are reliable.

In conclusion, although certain limitations still exist in this meta-analysis, the conclusions we obtained currently support that there exists a significant positive correlation between soluble *α*-Klotho and eGFR in patients with CKD. Also, a significant negative correlation between *α*-Klotho and FGF23 levels is found. This raises hope to employ *α*-Klotho and FGF23 as candidates of biomarkers of CKD patients with high sensitivity and specificity, preceding decline dramatically with different CKD stages and disturbance of CKD-mineral metabolism. However a large prospective cohort study and basic research are still in urgent needs to consolidate our conclusions and verify the following: (a) a detailed mechanism that kidney how to maintain *α*-Klotho homeostasis; (b) FGF-23 upregulation, *α*-Klotho deficiency, which occurs first; (c) whether replacement therapy (exogenous and endogenous) is safe and clinically effective.

## Figures and Tables

**Table 1:** Inclusion and exclusion criteria.

**Inclusion criteria**	(1) Investigation of the relationship between soluble *α*-klotho (measured in plasma or serum) and renal function associated indexes (eGFR was determined based on CKD-EPI or MDRD) in chronic kidney disease using correlation analyses.
	(2) Enrolling adults (mean age ≥18 years old).
	(3) Containing complete data information.
	(4) Research article published in the peer-reviewed journals.

**Exclusion criteria**	(1) Examination of the correlation between renal Klotho (mRNA or protein expression) and other related parameters.
	(2) Studies that didn't contain correlation coefficients (R value) between renal function associated indexes in CKD and soluble *α*-klotho protein.
	(3) Special populations such as those who suffered from renal replacement therapy or study populations with one specific kidney disease, etc. Diabetic nephrology patients were excluded.
	(4) Animal-based experiments, in-vitro experiments, having no insufficient data, having significant overlap in the study populations, all duplicated publications, or enrolling only children
	(5) Case reports, posters, reviews and meeting abstracts

**Table 2:** Characteristics of included studies.

Study	Publish year	Nation	Average age	N	index	Correlation coefficient	Study design	Assay utilization	Intact/C-terminal of FGF-23
Masashi Kitagawa	2013	Japan	58	114	GFR	0.3913*∗*	Cross-sectional	ELISA(Japan)	Intact
					Age	-0.272*∗*			
					FGF23	-0.1751*∗*			
					Ca	0.1618*∗*			
					Phos	-0.3034*∗*			

Michishige Ozeki	2014	Japan	68.9	185	GFR	0.209*∗*	Retrospective	ELISA(Japan)	Intact
					FGF23	-0.14			
					Ca	0.06*∗*			
					Phos	0.05			

Tetsu Akimoto1	2012	Japan	56	131	GFR	0.232	Cross-sectional	ELISA(Japan)	Nr
					Age	-0.172			

Hyoung Rae Kim	2013	Korea	45.7	243	GFR	0.502	Cross-sectional	ELISA(Japan)	Intact
					Age	-0.395			
					FGF23	-0.245			
					Ca	0.257			
					Phos	-0.169			

Valerie Hage	2014	France	46.7	60	GFR	0.11	Cross-sectional	ELISA(Japan)	Nr
					FGF23	0.23			

Sarah Seiler	2012	Germany	65.5	321	FGF23	-0.03*∗*	Prospective	ELISA(Japan)	C-terminal
					Ca	0.01*∗*			
					Phos	-0.06*∗*			

Ivana Pavik	2013	Switzerland	52.7	87	GFR	0.64	Cross-sectional	ELISA(Japan)	C-terminal

Yoshiko Shimamura	2012	Japan	63.8	292	GFR	0.441	Prospective	ELISA(Japan)	Intact
					Age	-0.345			
					FGF23	-0.156			

Alexandra Scholze	2014	Denmark	68	24	GFR	0.11*∗*	RCT	ELISA(Japan)	Intact
					Age	-0.25*∗*			
					FGF23	0.09*∗*			
					Phos	-0.10*∗*			

*Note. ∗*spearman correlation coefficient, otherwise, Pearson correlation coefficient. Nr representative not reported.

## Supplementary Materials

Supplementary Materials Supplementary Materials Figure 1. To detect the study bias, we conducted funnel plot of the publication bias for *α*-Klotho and eGFR group. The result is suggestive of an indistinctive small study bias. Supplementary Materials Figure 2. To test whether a study has a significant impact on the results, sensitivity estimates for *α*-Klotho and eGFR group with corresponding 95% CIs were conducted. In the sensitivity analysis, the pooled RR ranged from 0.32 (95%CI, 0.19~0.44) to 0.37 (95%CI, 0.26~0.48). No study had a significant impact on the total combined results. Supplementary Materials Figure 3. To detect the study bias, we conducted funnel plot of the publication bias for *α*-Klotho and FGF-23 group. The result is suggestive of an indistinctive small study bias. Supplementary Materials Figure 4. To test whether a study has a significant impact on the results, sensitivity estimates for *α*-Klotho and FGF-23 group with corresponding 95% CIs were conducted. In the sensitivity analysis, the pooled RR ranged from -0.13 (95%CI, -0.20~-0.06) to -0.07(95%CI, -0.17~-0.02). No study had a significant impact on the total combined results. Supplementary Materials Figure 5. To detect the study bias, we conducted funnel plot of the publication bias for *α*-Klotho and Ca group. The result is suggestive of an indistinctive small study bias. Supplementary Materials Figure 6. To test whether a study has a significant impact on the results, sensitivity estimates for *α*-Klotho and Ca group with corresponding 95% CIs were conducted. In the sensitivity analysis, the pooled RR ranged from 0.05 (95%CI, -0.03~0.13) to 0.16 (95%CI, 0.04~0.28). No study had a significant impact on the total combined results. Supplementary Materials Figure 7. To detect the study bias, we conducted funnel plot of the publication bias for *α*-Klotho and PTH group. The result is suggestive of an indistinctive small study bias. Supplementary Materials Figure 8. To test whether a study has a significant impact on the results, sensitivity estimates for *α*-Klotho and PTH group with corresponding 95% CIs was conducted. In the sensitivity analysis, the pooled RR ranged from -0.22 (95%CI, -0.33~-0.12) to -0.07 (95%CI, -0.30~0.17). No study had a significant impact on the total combined results. Supplementary Materials Figure 9. To detect the study bias, we conducted funnel plot of the publication bias for *α*-Klotho and PHOS group. The result is suggestive of an indistinctive small study bias. Supplementary Materials Figure 10. To test whether a study has a significant impact on the results, sensitivity estimates fo *α*-Klotho and PHOS group with corresponding 95% CIs were conducted. In the sensitivity analysis, the pooled RR ranged from -0.08 (95%CI, -0.19~0.08) to -0.00 (95%CI, -0.09~0.08). No study had a significant impact on the total combined results.
