# Supplementary material for: Correlation between Soluble α-Klotho and Renal Function in Patients with Chronic Kidney Disease: A Review and Meta-Analysis
Source: Biomed Res Int. 2018 Aug 12;2018:9481475. doi: 10.1155/2018/9481475 (PMC6109492; doi:10.1155/2018/9481475)
Supplement: Supplementary Materials — Figure 1. To detect the study bias, we conducted funnel plot of the publication bias for α-Klotho and eGFR group. The result is suggestive of an indistinctive small study bias. Supplementary Materials Figure 2. To test whether a study has a significant impact on the results, sensitivity estimates for α-Klotho and eGFR group with corresponding 95% CIs were conducted. In the sensitivity analysis, the pooled RR ranged from 0.32 (95%CI, 0.19~0.44) to 0.37 (95%CI, 0.26~0.48). No study had a significant impact on the total combined results. Supplementary Materials Figure 3. To detect the study bias, we conducted funnel plot of the publication bias for α-Klotho and FGF-23 group. The result is suggestive of an indistinctive small study bias. Supplementary Materials Figure 4. To test whether a study has a significant impact on the results, sensitivity estimates for α-Klotho and FGF-23 group with corresponding 95% CIs were conducted. In the sensitivity analysis, the pooled RR ranged from -0.13 (95%CI, -0.20~-0.06) to -0.07(95%CI, -0.17~-0.02). No study had a significant impact on the total combined results. Supplementary Materials Figure 5. To detect the study bias, we conducted funnel plot of the publication bias for α-Klotho and Ca group. The result is suggestive of an indistinctive small study bias. Supplementary Materials Figure 6. To test whether a study has a significant impact on the results, sensitivity estimates for α-Klotho and Ca group with corresponding 95% CIs were conducted. In the sensitivity analysis, the pooled RR ranged from 0.05 (95%CI, -0.03~0.13) to 0.16 (95%CI, 0.04~0.28). No study had a significant impact on the total combined results. Supplementary Materials Figure 7. To detect the study bias, we conducted funnel plot of the publication bias for α-Klotho and PTH group. The result is suggestive of an indistinctive small study bias. Supplementary Materials Figure 8. To test whether a study has a significant impact on the results, [file 9481475.f1.zip › 9481475.f1/Supplementary Materials Table 1_BMRI_2422654.doc]

Supplementary Materials Table 1. Subgroup analysis

| Index | Subgroups | *No. of studies* | *Sample size* | *Correlation Coefficient(95%CI)* | *P for heterogeneity* | *P for group heterogeneity* |
| --- | --- | --- | --- | --- | --- | --- |
| eGFR | Nation | 8 |  | 0.37(0.31, 0.43) | 0.002 | 0.939 |
|  | Asia | 5 | 965 | 0.37(0.30, 0.43) | 0.021 |  |
|  | Europe | 3 | 171 | 0.37(0.22, 0.53) | 0.005 |  |
|  | Age | 8 |  | 0.37(0.31, 0.43) | 0.002 | 0.274 |
|  | <60 | 5 | 635 | 0.40(0.32, 0.48) | 0.005 |  |
|  | ≥60 | 3 | 501 | 0.33(0.24, 0.42) | 0.044 |  |
|  | eGFR | 8 |  | 0.37(0.31, 0.43) | 0.002 | 0.467 |
|  | ≥45 | 6 | 820 | 0.35(0.29, 0.42) | 0.001 |  |
|  | <45 | 2 | 316 | 0.40(0.29, 0.51) | 0.164 |  |
| FGF-23 | Nation | 7 |  | -0.11(-0.17, -0.06) | 0.032 | 0.002* |
|  | Asia | 4 | 834 | -0.17(-0.24, -0.11) | 0.001 |  |
|  | Europe | 3 | 405 | 0.01(-0.09, 0.11) | 0.806 |  |
|  | Age | 7 |  | -0.11(-0.17, -0.06) | 0.032 | 0.306 |
|  | <60 | 3 | 417 | -0.15(-0.25, -0.06) | 0.002 |  |
|  | ≥60 | 4 | 822 | -0.09(-0.16, -0.02) | 0.008 |  |
|  | eGFR | 7 |  | -0.11(-0.17, -0.06) | 0.032 | 0.239 |
|  | ≥45 | 4 | 602 | -0.15(-0.23, -0.07) | 0.022 |  |
|  | <45 | 3 | 637 | -0.08(-0.16, -0.00) | 0.249 |  |

* *P*<0.05 represents statistical significance
